# Angiogenesis, Metabolism, Endothelial and Platelet Markers in Diabetes and Cardiovascular Disease

**DOI:** 10.3389/bjbs.2022.10313

**Published:** 2022-03-22

**Authors:** A. D. Blann, J. E. Brown, R. Heitmar

**Affiliations:** ^1^ School of Applied Sciences, Huddersfield University, Huddersfield, United Kingdom; ^2^ Department of Biosciences, College of Health and Life Sciences, Aston University, Birmingham, United Kingdom

**Keywords:** angiogenesis, endothelial cells, platelets, diabetes, cardiovascular diesease

## Abstract

**Introduction:** Diabetes is a leading risk factor for cardiovascular disease (CVD), the pathophysiology of both being linked to metabolic, endothelial, renal, angiogenic and platelet abnormalities. We hypothesised that abnormalities in these systems are more adverse in those whose CVD is compounded by diabetes, compared to those with diabetes or CVD alone.

**Materials and methods:** Serum or plasma from 66 patients with diabetes alone, 76 with CVD alone, and 70 with both diabetes and CVD i.e. diabetic cardiovascular disease, was probed for markers of angiogenesis [angiopoietin 1 and 2, vascular endothelial growth factor (VEGF) and endoglin], metabolic [soluble receptor for advanced glycation products (sRAGE), leptin, lipocalin-2, interleukin-8, and cystatin-C], the endothelium (von Willebrand factor, endothelial microparticles and soluble E selectin)], and the platelet (platelet microparticles and soluble P selectin) by ELISA, Luminex or flow cytometry.

**Results:** VEGF (*p* = 0.04), von Willebrand factor (*p* = 0.001) and endothelial microparticles (*p* = 0.042) were all higher in diabetic cardiovascular disease than in diabetes alone and cardiovascular disease alone. Soluble E selectin was higher in diabetic cardiovascular disease than in diabetes alone (*p* = 0.045), whilst cystatin-C (*p* = 0.004) and soluble P selectin (*p* < 0.001) were higher in diabetes and diabetic cardiovascular disease than in cardiovascular disease alone. There were no differences in angiopoietin 1 or 2, endoglin, sRAGE, leptin, lipocalin-2, or interleukin-8.

**Conclusion:** Angiopoietin 1 or 2, endoglin, sRAGE, leptin, lipocalin-2, interleukin-8, and cystatin-c cannot differentiate diabetes from cardiovascular disease, or both conditions combined. Our data point to a more adverse endothelial (von Willebrand factor, endothelial microparticles), and angiogenic profile (VEGF) in those with diabetic cardiovascular disease, supporting the view that this group should be targeted more aggressively.

## Introduction

Cardiovascular disease (CVD) has a complex pathogenesis and can manifest as coronary artery disease (such as previous myocardial infarction and coronary artery stenosis/occlusion), heart failure, cerebrovascular disease (leading to stroke) and peripheral artery disease (often requiring amputation). Diabetes is a major risk factor for CVD, but many diabetics also have other risk factors and other pathology such as retinopathy, obesity, hypertension, and renal disease, several of which are also present in CVD (1, 2). From a perspective of clinical practice, CVD, where by definition a major event has already occurred and/or is present, requires more complex and urgent management, often focussing on risk factors and signs/symptoms. The pathophysiology of CVD and diabetes involves many different disease processes, such as inflammation and those acting on the endothelium and the platelet, adverse changes to both potentially leading to thrombosis and hypertension, and a developing theme in both is inappropriate angiogenesis (3, 4).

Abnormalities in several other metabolic processes are evident in these diseases, and may be marked by molecular markers such as sRAGE (soluble receptor for advanced glycation end-products, arising from the endothelium and elsewhere, and with pathophysiological links to diabetes), cystatin-c (reflecting renal function), leptin (arising from adipose tissue and with roles in digestion), lipocalin-2 (an inflammatory adipokine, also known as neutrophil gelatinase-associated lipocalin, linked to nephropathy) and interleukin-8 (also known as CXCL8, a pro-inflammatory cytokine) (5–9). Leading soluble markers of angiogenesis include vascular endothelial growth factor (VEGF), angiopoietins 1 and 2 (Ang-1, Ang-2) and endoglin (CD105) (10–12), whilst pathophysiology of the endothelium may be marked by von Willebrand factor (vWf), soluble E-selectin (sEsel) and by endothelial microparticles (EMPs) (13–16). Changes in platelet pathophysiology may be reflected by altered soluble P-selectin and platelet microparticles (16, 17).

Despite these abnormalities, diabetes and CVD without diabetes are often viewed as having a similar pathogenicity with equivalent likelihoods of disease progression (if un- or poorly treated). However, the strength of the certainty of these pathogenic processes in the presence of both disease processes, and their combination, is unclear. We therefore hypothesised that those patients with both conditions would, despite appropriate clinical care, have a more adverse marker profile than those with either disease alone.

## Materials and Methods

We tested our hypothesis in 212 age and gender-matched patients with an established history of diabetes (*n* = 66), CVD (*n* = 76), or both conditions (*n* = 70) recruited from Out-patients attending City Hospital, Birmingham, United Kingdom. Our power calculation was based on a virtual test statistic with a mean of 100 or 105 units and a standard deviation of 20 units in the diabetes or CVD groups, a difference that is not statistically significant. We hypothesised a significant (*p* < 0.05 by Tukey’s post-hoc test) difference of 15 units (i.e., 75% of a standard deviation) between the diabetes and CVD groups and the diabetic CVD group. In order to defend the derived data we would need 60 patients per group, 180 overall In order to generate improved confidence, we recruited until we had at least 10% more subjects per group, which was ultimately 66, 76, and 70 per group. Inclusion criteria were type 2 diabetics attending a diabetes clinic, and patients with atherosclerotic coronary artery disease attending a cardiology clinic. Exclusion criteria were age <18 years, present or history of cancer, lone atrial fibrillation, lone heart failure, other endocrine or metabolic disease, and any inflammatory disease such as thyroiditis or rheumatoid arthritis. The study had the approval of the local research ethics committee (East Midland-Leicester, United Kingdom, 12/EM/0062) and informed written consent was obtained from all participants in accordance with the Declaration of Helsinki.

Each participant provided blood samples for routine and research analyses. Creatinine, urinary albumin to creatinine ratio, and HbA1c were measured by standard routine methods of the Hospital Pathology Laboratory. Blood pressure was measured by an Omron M3 digital sphygmomanometer (Omron Healthcare Ltd., Milton Keynes, United Kingdom). Research indices Ang-1, Ang-2, interleukin-8, sRAGE, Cystatin C, leptin, lipocalin, endoglin, VEGF and C-reactive protein (CRP) were analysed using MAGPIX Luminex assays (Merck Millipore, Burlington, Mass, United States) that uses a miniaturised liquid array immunoassay with colour-coded magnetic microspheres. Briefly, 20 µL samples of diluted (1/5) plasma were analysed using xMAP Luminex technology and xPONENT software (Luminex, Austin, Texas, United States). vWf, sEsel (both in serum) and sPel (in citrated plasma) were determined by ELISA using commercial reagents (Dako, Ely, United Kingdom and R&D Systems, Abingdon United Kingdom). PMPs and EMPs were measured in citrated plasma by fluoresence flow cytometry using monoclonal antibodies to CD42b and CD144 respectively (Abcam, Cambridge, United Kingdom) as described elsewhere (18, 19).

Continuously variable data are presented as mean with standard deviation or median with interquartile range and analysed by analysis of variance or the Kruskal-Wallis test as distribution demands. Categorical data is presented as number and percentage and analysed by the chi-squared test. Differences between groups were sought by Tukey’s post-hoc test. Those indices significantly different in univariate analyses were further analysed by multivariate logistic regression. Correlations were sought using Spearman’s method. *p* < 0.05 was considered significant, analyses were performed on Minitab 19 (Coventry, United Kingdom).

## Results

Table 1 shows standard clinical, laboratory and demographic data on the three groups of patients gave the expected raised BMI and HbA1c in diabetes. Diastolic blood pressure was lower in those with both diseases compared with either alone, perhaps linked to the greater use of anti-hypertensive agents. Although creatinine was higher in those with any diabetes, the eGFR was no different, perhaps reflecting age, gender, and racial profile of the groups. Greater use of statins in those with any CVD most likely reflects guidance from the UK’s National Institute for Health and Care Excellence (20), a leading document regarding clinical practice. Notably, the median and upper quartile CRP in each group was less than the local reference range of <5 mg/L.

**TABLE 1:** Demographic, laboratory, and clinical data.

	Diabetes (*n* = 66)	Cardiovascular disease (*n* = 76)	Both diabetes and cardiovascular disease (*n* = 70)	*p* value
Demographics				
Age (years)	62.8 (10.3)	63.0 (10.7)	64.8 (8.2)	0.409
Gender (M/F)	41/25	51/25	53/17	0.224
Clinical data				
SBP (mm Hg)	143 (18)	136 (20)	137 (21)	0.064
DBP (mm Hg)	79 (12)	79 (13)	73 (12)	0.008
BMI (kg/m^2^)	31.4 (6.2)	27.0 (4.2)	30.3 (6.3)	<0.001
Coronary artery disease (*n*, %)	—	57 (75%)	56, (80%)	0.470
Peripheral artery disease (*n*, %)	—	10 (13.1%)	8 9 (11.4%)	0.101
Cerebrovascular disease (*n*, %)	—	15 (19.7%)	6 (8.6%)	0.055
Laboratory data				
HbA1c (mmol/mol)	59 (15)	42 (3)	64 (23)	<0.001
Creatinine (µmol/L)	106 (45)	88 (21)	100 (30)	0.006
Estimated GFR	66 (21)	72 (15)	67 (19)	0.118
Urinary ACR	4.7 [1.1–14.8]	—	3.1 [1.4–16.0]	0.973
CRP (mg/L)	3.1 [2.5–3.5]	3.0 [2.6–3.5]	3.0 [2.7–3.5]	0.945
Antithrombotic medications				
Anti-platelet (*n*, %)	23 (34.8%)	65 (85.6%)	58s (82.9%)	<0.001
Anti-coagulant (*n*, %)	6 (9.1%)	8 (10.5%)	5 (7.1%)	n.a.
Dual therapy (*n*, %)	0	1 (1.3%)	5 (7.1%)	n.a.
Neither therapy (*n*, %)	37 (56.1%)	2 (2.6%)	2 (2.9%)	n.a.
Anti-hypertensive medications				
Calcium channel blocker (*n*, %)	37 (56.1%)	23 (30.3%)	27 (38.6%)	0.007
ACEI/ARB (*n*, %)	42 (63.6%)	55 (72.4%)	57 (81.4%)	0.066
Beta-blocker (*n*, %)	18 (27.3%)	33 (25.1%)	41 (58.6%)	0.001
Diuretic (*n*, %)	32 (48.5%)	16 (21.1%)	35 (50.0%)	<0.001
Lipid-lowering medications				
Statin (*n*, %)	46 (69.7%)	72 (94.7%)	68 (97.1%)	<0.001
Resin (*n*, %)	0	1 (1.3%)	1 (1.4%)	n.a.
Glucose-regulating medications				
Metformin (*n*, %)	49 (74.2%)	—	41 (58.6%)	0.053
Insulin (*n*, %)	28 (42.4%)	—	26 (37.1%)	0.529
Sulphonylurea (*n*, %)	13 (19.7%)	—	14 (20.0%)	0.964
DPP-4 inhibitor (*n*, %)	15 (22.7%)	—	11 (15.7%)	0.298

SBP, systolic blood pressure; DBP, diastolic blood pressure; BMI, body mass index; GFR, glomerular filtration rate; ACR, albumin to creatinine ratio; CRP, C-reactive protein; ACEI/ARB, angiotensin converting enzyme inhibitor/angiotensin receptor blocker; Data mean (SD), *n*, %, or median [interquartile range]; n.a., not analysable (underpowered). *p* values by *t* test or Mann-Whitney for two groups, ANOVA, or Kruskal-Wallis for three group, categorical data by chi-squared.

Table 2 shows research markers. Levels of VEGF, but not other markers of angiogenesis, were higher in those with both diabetes and CVD compared to those with diabetes alone (*p* = 0.012). Levels of endoglin correlated weakly with those of angiopoietin-1 (r = 0.21, *p* = 0.007). In multivariate logistic regression only VEGF remained an independent predictor of the presence of diabetes and CVD. Of the metabolic markers, only cystatin-C differed between the groups, being higher in diabetes plus CVD than in CVD alone (both *p* = 0.001). Overall, levels of cystatin-C correlated modestly with those of creatinine (r = 0.45, *p* < 0.001) and eGFR (r = −0.49 *p* < 0.001). In logistic regression, both creatinine (*p* = 0.034) and cystatin-C (*p* = 0.009) were independent predictors of CVD versus diabetes plus CVD. Overall, leptin correlated modestly with HbA1c (r = 0.31, *p* = 0.02) and BMI (r = 0.45, *p* < 0.001).

**TABLE 2:** Research indices.

	Diabetes (*n* = 66)	Cardiovascular disease (*n* = 76)	Both diabetes and cardiovascular disease (*n* = 70)	*p* value
Angiogenesis markers				
Angiopoietin-1 (ng/ml)	3.0 [1.6–4.4]	2.7 [1.8–4.2]	2.7 [1.5–4.0]	0.716
Angiopoietin-2 (ng/ml)	0.77 [0.58–1.62]	0.73 [0.49–1.16]	0.90 [0.62–1.40]	0.124
VEGF (pg/ml)	21 [13–36]	23 [13–37]	31 [17–61]	0.040
Endoglin (ng/ml)	1.12 [0.81–1.67]	1.18 [0.84–1.65]	1.01 [0.80–1.46]	0.421
Metabolic markers				
sRAGE (ng/ml)	1.6 [1.2–2.6]	1.7 [1.4–2.6]	1.7 [1.2–2.6]	0.916
Cystatin-C (ng/ml)	21 [16–25]	18 [16–22]	22 [18–27]	0.004
Leptin (ng/ml)	1.7 [0.8–3.4]	1.4 [0.7–2.9]	1.9 [0.9–3.5]	0.381
Lipocalin (ng/ml)	57 [42–73]	67 [39–84]	64 [46–77]	0.310
Interleukin-8 (ng/ml)	0.17 (0.03)	0.18 (0.02)	0.17 (0.02)	0.736
Endothelial and platelet markers				
vWf (IU/dl)	109 [22]	112 [23]	123 [25]	0.001
sEsel (ng/ml)	24 [15–29]	25 [22–32]	27 [21–31]	0.045
EMPs (×10^3^/µL)	30 [7–88]	32 [4–97]	53 [17–112]	0.042
sPsel (ng/ml)	20 [13–39]	15 [13–18]	18 [15–26]	<0.001
PMPs (×10^3^/µL)	6 [1–42]	11 [1–55]	9 [1–38]	0.603

Data mean (SD) or median [inter-quartile range]. VEGF, vascular endothelial growth factor; sRAGE, soluble receptor for advanced glycation products, vWf, von Willebrand factor, sEsel, soluble E selectin, sPsel, soluble P selectin, EMPs, endothelial microparticles; PMPs, platelet microparticles. *p* value by ANOVA, or Kruskal-Wallis.

There were several differences in endothelial and platelet markers. vWf and EMPs were both higher in diabetes plus CVD compared to diabetes alone (*p* = 0.001 and *p* = 0.045 respectively) and in diabetes plus CVD versus CVD alone (*p* = 0.007 and *p* = 0.02, respectively. However, these two markers failed to correlate significantly (r = 0.04, *p* = 0.53). sEsel was higher in CVD alone (*p* = 0.038) and in diabetes plus CVD (*p* = 0.027) compared to diabetes alone. In logistic regression, both vWf (*p* < 0.001) and EMPs (*p* = 0.029) were significant independent predictors of group. There was no overall difference in PMPs, but levels of sPsel were higher in diabetes alone (*p* < 0.001) and in diabetes plus CVD (*p* = 0.001) compared to CVD alone.

## Discussion

Diabetes is perhaps the leading risk factor for CVD, and accordingly requires focussed management to clinically-relevant targets (20–22), whilst the importance of concurrent diabetes and CVD is also becoming recognised (23, 24). Although intensive blood glucose control reduces the risk of myocardial infarction, it does not reduce all-cause or cardiovascular mortality (25), implying other measures targeting alternative risk factors/pathology are required.

We tested the hypothesis that patients with CVD compounded by diabetes would have more adverse metabolic, angiogenic, endothelial, platelet and renal profiles than those with either disease in isolation. Of the markers of angiogenesis, VEGF was marginally higher in those with diabetes and CVD. Neri et al have argued that increased VEGF in diabetes reflects subclinical endothelial dysfunction (26), whilst others suggest it is the consequence of hypoxia (27, 28). Raised VEGF in our patients may be a marker of early diabetic retinopathy (29, 30), although this clinical feature was not recorded. Increased levels of this growth factor are widely regarded as reflective of aberrant angiogenesis and as possible markers of endothelial pathology (26–30). Angiopoietins 1 and 2 may also have a role in diabetic retinopathy (31, 32), and increased levels of these molecules have been described in acute myocardial infarction (33). A report of similar design to ours with diabetes and unstable angina pectoris (UAP) found no changes in angiogenic factors in diabetes alone, but increased VEGF and angiopoietin-2 in UAP. Sub-group analysis of the latter according to diabetes failed to find differences in any angiogenic markers, whereas we report increased VEGF in diabetes plus CVD compared to CVD alone, perhaps as our study has around twice the statistical power (34). Increased levels of endoglin may have a role in diabetic retinopathy, hypertension, and the metabolic syndrome (35–37), but in our hands it could not differentiate any of the diabetes and CVD groups. Although described as an endothelial product (38, 39), levels failed to correlate with any established markers of this cell.

Of the metabolic markers, only cystatin-C differed between the groups, being highest in both diabetes groups, and correlating overall with renal markers. We speculate that it may have an as yet unspecified role in these conditions, perhaps linked to renal function. Notably, in a prospective study, high levels of cystatin-C brought a hazard ratio of 1.66 for CVD death (40). Although leptin failed to differ between the groups, in the entire cohort it correlated strongly with BMI, possibly reflecting its adipocyte origin, but less convincingly with HbA1c. Whilst a precise pathophysiological role(s) for this molecular is unclear, increased levels predict prognosis in established coronary atherosclerosis, even when adjusting for BMI (41).

All endothelial markers differed between the groups, the largest being in vWf, where levels were some 10% higher in diabetes plus CVD, supporting the broad hypothesis of increased vascular damage in the face of multiple risk factors (42, 43). Although the relative increase in EMPs (∼70%) was considerably higher than that of vWf, a greater variance brought only marginal significance. Nevertheless, increased EMPs are likely to have some pathological significance independent of vWf as the two failed to correlate. sPsel levels again tracked both diabetes groups, an observation that cannot be ascribed to the increased use of aspirin (known to reduce sPsel (44)) in our patients with CVD. This is in contrast to a previous report (45) which found no difference in sPsel in diabetes compared to diabetes plus CVD, although an improvement is risk factor profiles with intensive treatment in diabetics alone resulted in reduced levels.

We acknowledge the limitation of a modest sample size, but consider a strength to be the real world nature of the patients managed in secondary care. Furthermore, the three groups are matched for CRP, and as levels are low the changes we report cannot be reflective of sub-clinical or laboratory defined inflammation.

## Conclusion

Our data supports the hypothesis that diabetes plus CVD brings a more adverse plasma marker profile than neither group alone. Indeed, levels of 8 of the 14 circulating research markers were highest in this combined group, despite apparent best clinical practice. In this group, increased VEGF may reflect inappropriate angiogenesis, whilst increased vWf, EMPs and soluble E selectin may be the result of further endothelial cell damage. If correct, this group is at highest risk of an adverse major cardiovascular event and so should be managed more aggressively.

## Data Availability

The original contributions presented in the study are included in the article/Supplementary Material, further inquiries can be directed to the corresponding author.
